# Integrated molecular and microenvironmental drivers of drug resistance in gastrointestinal cancers: mechanisms, immunotherapy challenges, and precision strategies

**DOI:** 10.3389/fonc.2025.1675745

**Published:** 2025-10-28

**Authors:** Heng Xu, Jiaan Lu, Jiangying Wu, Kangling Zhang, Xuancheng Zhou, Ziqi Gao, Jingqi Feng, Ziye Zhuang, Xiaolin Zhong

**Affiliations:** ^1^ Clinical Medical College, Southwest Medical University, Luzhou, China; ^2^ First Clinical Medical College, Guangdong Medical University, Zhanjiang, Guangdong, China; ^3^ Department of Gastroenterology, The Affiliated Hospital of Southwest Medical University, Luzhou, China

**Keywords:** cancer, drug resistance, tumor microenvironment (TME), molecular mechanisms, artificial intelligence (AI), precision oncology, drug sensitivity profiling, therapeutic strategies

## Abstract

Resistance to chemotherapy, targeted agents, and particularly immunotherapy remains the principal challenge in the management of gastrointestinal malignancies. This review aims to comprehensively delineate the molecular and microenvironmental drivers of resistance, with emphasis on mechanisms impacting immunotherapy response, and evaluate emerging, mechanism−guided interventions (including immunotherapeutic combinations) for precision therapy. We first examine intrinsic mechanisms—including drug−target alterations, dysregulated drug metabolism and efflux, hyperactivation of DNA damage repair pathways, and epigenetic remodeling—and extrinsic influences stemming from the tumor microenvironment and extracellular matrix remodeling. We then highlight epithelial–mesenchymal transition (EMT) as a critical nexus that integrates stromal cues with cell−intrinsic survival programs, thereby promoting drug efflux and immune evasion. Next, we discuss how single−cell and spatial omics, liquid biopsy, patient−derived organoids, and AI−enabled analytics facilitate subclone−level mapping of resistance networks and real−time tracking of clonal evolution. Finally, we review mechanism−based strategies—including KRAS G12C inhibitors, efflux−pump antagonists, apoptosis reactivators, and epigenetic/autophagy modulators—and propose an integrated, multimodal regimen leveraging immunotherapy where appropriate, informed by real-time drug sensitivity data (e.g., from liquid biopsy), dynamic biomarkers and AI−driven optimization to overcome resistance and improve patient outcomes.

## 1 Introduction

Gastrointestinal (GI) tumors comprise malignant neoplasms arising within the digestive system, including gastric carcinoma, colorectal carcinoma, hepatocellular carcinoma, pancreatic carcinoma, and gallbladder carcinoma (1). Their high incidence and mortality rates impose an escalating global public health burden (2, 3). Despite significant advances in surgical resection, chemotherapy, molecularly targeted therapies, and immunotherapy, drug resistance remains the principal obstacle to durable treatment response (4). Drug resistance—defined as a marked diminution in tumor sensitivity following therapy, leading to treatment failure and poorer prognosis—can be classified into primary (pre-existing) resistance and acquired resistance that emerges during the course of treatment; these forms often co-exist and interact to drive disease relapse and progression (5–7). Consequently, delineating resistance mechanisms in the gastrointestinal tract, particularly those constraining immunotherapeutic efficacy, is crucial. This study examines how integrating molecular profiling, immunotherapeutic data, and functional drug sensitivity assays can inform the design of enhanced treatment regimens to improve clinical outcomes. Critically, translating molecular insights into clinical action requires bridging two key gaps: predicting immunotherapy resistance driven by dynamic tumor-immune interactions, and quantifying drug sensitivity at the individual patient level. This review therefore emphasizes functional drug sensitivity profiling—using ex vivo models and liquid biopsy—as a linchpin for integrating molecular mechanisms with immunotherapeutic strategies to design adaptive treatment regimens.

## 2 Overview of anti−tumor drug resistance mechanisms in gastrointestinal tumors

**Figure 1:**
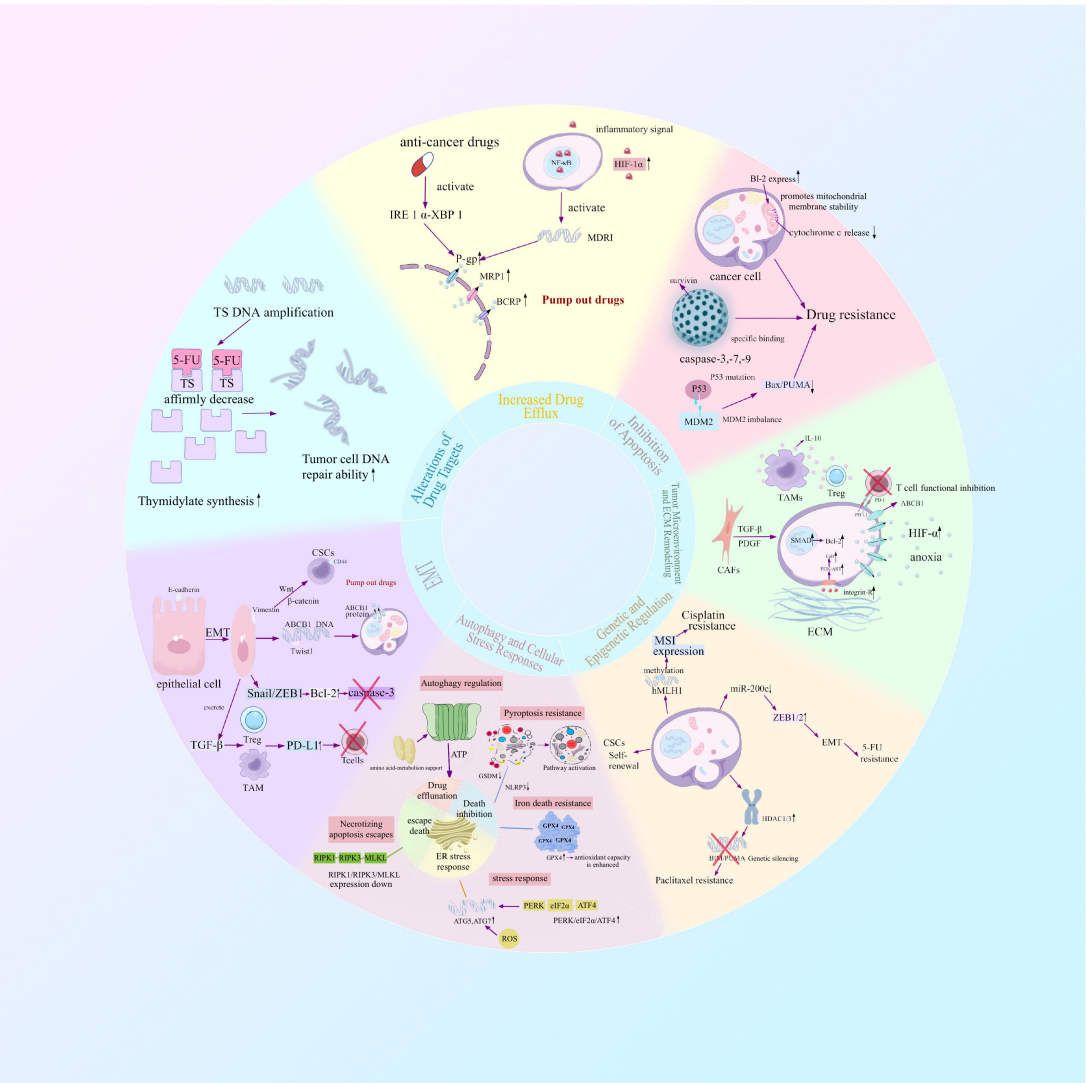
The development of therapeutic resistance in gastrointestinal cancers is a multifactorial phenomenon driven by both tumor cell–intrinsic programs and extrinsic influences from the surrounding microenvironment. Intrinsic mechanisms include alterations in drug targets, dysregulation of drug metabolism and efflux systems, overactivation of DNA damage repair pathways, and widespread epigenetic reprogramming. In parallel, extrinsic mechanisms arise from dynamic remodeling of the tumor microenvironment (TME) and extracellular matrix (ECM), which together establish physical and biochemical barriers that impede effective drug delivery. Moreover, epithelial–mesenchymal transition (EMT) and the acquisition of cancer stem cell (CSC)-like properties have emerged as central determinants of resistance phenotypes. EMT-associated transcription factors not only drive tumor cell invasiveness and migratory capacity but also endow cells with enhanced resistance to apoptosis. Simultaneously, CSC populations exhibit innate tolerance to chemotherapy and possess the ability to repopulate tumors post-treatment, thereby serving as a persistent reservoir of drug-refractory cells.

Acquired resistance develops when primary resistance is a measure of tumor cell insensitivity inherent to the tumor that occurs before any form of therapy, which causes suboptimal responses to initial chemotherapy or targeted therapies. Acquired resistance develops when once−sensitive cells, under sustained drug pressure, gain resistance through genetic mutations or adaptive reprogramming (8, 9). The emergence of resistance is a multifactorial process involving both cell−intrinsic and microenvironmental factors. Intrinsic mechanisms comprise drug target alterations, drug metabolism and efflux dysregulation, hyperactivation of DNA damage repair pathways, and epigenetic alterations (9–12). Extrinsic mechanisms are derived from the tumor microenvironment and extracellular matrix (ECM) remodeling, which together generate physical and biochemical barriers to drug delivery efficiency (13, 14). In addition, epithelial–mesenchymal transition (EMT) and the induction of cancer stem cell (CSC) features have been established as underlying determinants of drug resistance. EMT−associated transcription factors not only promote invasion and migration but also confer anti−apoptotic capacity, while CSC populations inherently tolerate drugs and regenerate the tumor mass after therapy, acting as reservoirs of resistant cells (15–18). These interconnected mechanisms synergize to establish the refractory phenotype of gastrointestinal tumors. Critically, these mechanisms also orchestrate immune evasion, constituting a major barrier to the efficacy of cancer immunotherapy, which has emerged as a pivotal therapeutic modality alongside chemotherapy and targeted agents (19) (**Figure 1**).

## 3 Resistance mechanisms

### 3.1 Intrinsic factors

#### 3.1.1 Functional drug sensitivity profiling platforms

Beyond mechanistic insights, patient-derived organoids (PDOs) serve as ex vivo micro-tumors for high-throughput drug screening. By exposing PDOs to chemotherapy, targeted agents, and immune checkpoint inhibitors (e.g., anti-PD-1), researchers quantify tumor-specific sensitivity and identify synergistic combinations (20). For instance, gastric cancer PDOs co-cultured with autologous T cells revealed that TGF-β blockade enhanced pembrolizumab efficacy in immunologically “cold” tumors (21). This functional approach complements genomic and immune profiling to prioritize clinically actionable regimens.

#### 3.1.2 Alterations of drug targets

Structural or expression modification of drug targets dramatically lowers drug–target affinity, hence, driving drug resistance. For instance, 5-fluouracil (5-FU) and raltitrexed are thymidylate synthase (TS) inhibitors that inhibit thymidine synthesis and DNA replication and repair, yet amplification or point mutations of the TS gene within gastrointestinal tumor cells generate TS overexpression or conformational changes which lower inhibitor affinity and induce drug resistance (22, 23). In a similar manner, resistance to anti-EGFR monoclonal antibodies (cetuximab, panitumumab) is largely caused by acquired mutations of the EGFR extracellular domain (ECD). S492R substitution abolishes cetuximab binding without impacting upon panitumumab affinity, with further ECD mutations (R451C, K467T, S464L, G465R, I491M) being present within clinical samples and cell−line models of resistance; it is these variants that modify antibody epitope conformation, allowing for continued EGFR (24–27). Moreover, mutations or amplifications of downstream effectors—KRAS, NRAS, BRAF, and PIK3CA—can activate the MAPK and PI3K−AKT pathways independently of EGFR, thus mediating both primary and acquired resistance to EGFR blockade (28, 29).

#### 3.1.3 Increased drug efflux

Overexpression of ATP-binding cassette (ABC) transporters is a key driver of drug resistance in gastrointestinal tumors. P-glycoprotein (P-gp; ABCB1), MRP1 (ABCC1), and BCRP (ABCG2) utilize ATP hydrolysis to actively extrude chemotherapeutic agents from the cytosol, markedly reducing intracellular drug accumulation and promoting resistance (30–34). Chemotherapy-induced endoplasmic reticulum stress has been shown to activate the IRE1α–XBP1 axis, leading to upregulation of ABCB1, ABCC1, and ABCG2 expression (35). In the gastric tumor microenvironment, inflammatory cues activate NF-κB and HIF-1α, which cooperatively bind the MDR1 promoter and drive P-gp overexpression, thereby conferring resistance to paclitaxel and doxorubicin. Overexpression of MRP1 is associated with reduced sensitivity to irinotecan and cisplatin (36, 37). Small molecule inhibitors of ABC transporters or upstream regulators are therefore potential options for reversing drug resistance and reestablishing chemosensitivity.

#### 3.1.4 Inhibition of apoptosis

Antineoplastic agents induce tumor cell death primarily via two canonical apoptotic cascades: the intrinsic, mitochondria-mediated pathway and the extrinsic, death receptor-mediated pathway. The intrinsic pathway involves Bax/Bak oligomerization, mitochondrial outer membrane permeabilization, cytochrome c release, and sequential activation of caspase-9 and caspase-3. The extrinsic pathway is initiated when ligands such as FasL or TRAIL bind their cognate death receptors, triggering caspase-8 and downstream caspase-3 activation.

Gastrointestinal tumor cells commonly evade both cascades through multiple mechanisms. First, anti-apoptotic Bcl-2 family members (Bcl-2, Bcl-xL, Mcl-1) are frequently overexpressed in gastric and colorectal cancers; by antagonizing Bax/Bak oligomerization and preserving mitochondrial integrity, they prevent cytochrome c release and caspase activation, thereby conferring resistance to 5-fluorouracil and oxaliplatin (38–40). Second, inhibitors of apoptosis proteins (IAPs), notably XIAP and Survivin, bind directly to caspase-3, -7, and -9, blocking their protease activity. Survivin expression is markedly elevated in gastric and colorectal tumors and correlates with resistance to cisplatin and irinotecan (41–46). Finally, dysfunction of the p53 pathway—via MDM2 overexpression or TP53 mutation—impairs transcriptional induction of pro-apoptotic targets such as Bax and PUMA, substantially reducing tumor cell sensitivity to DNA-damaging agents (47–54).

#### 3.1.5 Genetic and epigenetic regulation

Genetic and epigenetic mechanisms regulate gene expression through reversible chemical modifications and non-coding RNAs without altering the underlying DNA sequence, playing a pivotal role in the development of drug resistance in gastrointestinal (GI) tumors. DNA methylation, particularly hypermethylation of gene promoters, leads to the silencing of tumor suppressor genes and drug-metabolizing enzymes, both of which contribute to resistance. For example, promoter hypermethylation of the mismatch repair gene MLH1 in gastric cancer results in microsatellite instability (MSI), thereby reducing the efficacy of platinum-based chemotherapeutics (55, 56).

Histone modifications such as acetylation, methylation, and phosphorylation exert their action by modifying chromatin structure to thereby control the expression of resistance-associated genes (57). In gastric cancer, overexpression of HDAC1/3 contributes to deacetylation, silencing pro-apoptotic genes such as BIM and PUMA, which is a key mechanism in taxane resistance (58). Additionally, noncoding RNAs such as microRNAs (miRNAs), and long noncoding RNAs (lncRNAs), regulate drug susceptibility by targeting mRNA or affecting signaling pathways of vital importance and drug-resistant clone expansion in the case of therapeutic pressure (59). For example, downregulation of the miR−200 family, particularly miR-200c, upregulates ZEB1/2 and promotes epithelial–mesenchymal transition (EMT), a process that enhances resistance to 5−fluorouracil (5−FU) in colorectal cancer (60–64). The epigenetic network also plays a critical role in maintaining the self-renewal and multi−lineage differentiation potential of cancer stem cells (CSCs), thereby supporting the persistence and expansion of drug-resistant clones under therapeutic stress. The network is a dynamic and adaptive system both promoting resistance and tumor growth and is thus of particular importance as a target in GI oncology (61, 65–67).

#### 3.1.6 Autophagy and cellular stress responses

Tumor cells orchestrate multiple modes of cell death regulation to collectively contribute to therapeutic resistance. Autophagy, a lysosome-mediated degradation pathway, maintains cellular homeostasis under nutrient deprivation, hypoxia, or drug-induced stress. In gastrointestinal (GI) cancers, autophagy acts in concert with stress responses—such as endoplasmic reticulum (ER) stress and oxidative stress—to promote survival and resistance under chemotherapy or targeted therapy pressure (68). The autophagic degradation of macromolecules generates ATP, which fuels drug efflux mediated by ATP-binding cassette (ABC) transporters, while the recycled amino acids and fatty acids fulfill the metabolic demands of tumor cells under treatment, thereby reinforcing chemoresistance. Conversely, defective autophagy leads to the accumulation of p62/SQSTM1, which activates NF-κB signaling and upregulates pro-survival and pro-inflammatory genes, exacerbating cisplatin resistance (69–71).

Ferroptosis is an iron-dependent, lipid peroxidation–driven form of cell death. GI tumor cells commonly resist ferroptosis inducers (e.g., erastin, RSL3) and certain chemotherapeutics by upregulating glutathione peroxidase 4 (GPX4), thereby enhancing antioxidant capacity (72–74). In addition, tumor cells evade necroptosis, a RIPK1/RIPK3/MLKL-dependent death pathway, through downregulation of RIPK3 or MLKL expression, or by exploiting molecular chaperones to suppress pathway activation, enabling escape from TNFα family cytokines or certain chemotherapy-induced cell death (75, 76). Pyroptosis, defined as gasdermin-mediated inflammatory lytic death, is also suppressed by tumor cells through downregulation of GSDM proteins (e.g., GSDMD, GSDME) or inhibition of upstream inflammasome activation (e.g., NLRP3), conferring resistance to immunotherapy- or chemotherapy-induced pyroptosis (77, 78).

Stress responses are pervasive during chemotherapy. Chemotherapy-induced ER stress activates the PERK/eIF2α/ATF4 axis, upregulating core autophagy genes (ATG5, ATG7), thereby enhancing autophagic flux, alleviating protein-folding stress, and supporting cell survival. Meanwhile, chemotherapy-induced reactive oxygen species (ROS) further activate the transcription factor Nrf2, driving the expression of antioxidants (HO-1, NQO1) and autophagy-related genes, ultimately increasing tolerance to oxidative and drug-induced damage.

### 3.2 Extrinsic factors

#### 3.2.1 Tumor microenvironment and ECM remodeling

The tumor microenvironment (TME) comprises cancer-associated fibroblasts (CAFs), immune and endothelial cells, along with extracellular matrix (ECM) components, cytokines, and metabolites; its dynamic network profoundly influences therapeutic response in gastrointestinal tumors (79–82). CAF-derived factors such as TGF-β and PDGF activate SMAD, PI3K/AKT and MAPK signaling, leading to upregulation of anti−apoptotic proteins (e.g., Bcl-2, Survivin) and enhanced resistance to 5-fluorouracil and oxaliplatin in colorectal cancer (83–86). Hypoxia within the TME stabilizes HIF-1α, which drives expression of ABC transporters (ABCB1, ABCG2), promoting drug efflux and metabolic reprogramming that confer a survival advantage under treatment pressure (87–90). Concurrently, immunosuppressive populations—including regulatory T cells (Tregs) and tumor−associated macrophages (TAMs)—together with upregulated immune checkpoint molecules such as PD-L1, inhibit cytotoxic T-cell activity, thereby contributing significantly to resistance against both chemotherapy and immunotherapy (91). Remodeling of the ECM—characterized by deposition of hyaluronan and laminin—increases interstitial fluid pressure, impedes drug penetration, and engages integrin/FAK signaling to promote tumor cell adhesion, survival, and migration, thereby exacerbating resistance phenotypes (92–94).

#### 3.2.2 Metabolomics analysis

Metabolomics, through the systematic profiling of dynamic changes in small-molecule metabolites, offers a unique lens for deciphering the mechanisms underlying drug resistance in gastrointestinal (GI) cancers. Accumulating evidence indicates that resistant tumor cells reprogram key metabolic pathways—including energy metabolism, redox homeostasis, and nucleotide biosynthesis—or engage metabolite-mediated epigenetic regulation to evade chemotherapeutic cytotoxicity. For instance, in oxaliplatin-resistant colorectal cancer cells, the expression of hexokinase 2 (HK2) and lactate dehydrogenase A (LDHA) is markedly upregulated, thereby enhancing glycolytic flux and reducing intracellular drug accumulation (95–97). In gemcitabine-resistant pancreatic cancer models, increased expression of glutamate–cysteine ligase catalytic subunit (GCLC), the rate-limiting enzyme in glutathione (GSH) synthesis, promotes the clearance of chemotherapy-induced reactive oxygen species (ROS) and preserves redox homeostasis, ultimately protecting tumor cells (98, 99). Moreover, resistance to 5-fluorouracil (5-FU) in colorectal cancer has been linked to the overexpression of thymidylate synthase (TYMS) and increased dUTPase activity, which together drive competitive inhibition of the drug’s molecular target (100, 101).

### 3.3 Epithelial–mesenchymal transition

Epithelial–mesenchymal transition is a cellular reprogramming process in which epithelial tumor cells lose polarity and cell-cell adhesion while acquiring mesenchymal characteristics; hallmarks include downregulation of E-cadherin and upregulation of Vimentin, accompanied by enhanced migratory and invasive capacities that confer resistance to anticancer agents (102–106). EMT also promotes a cancer stem cell phenotype through activation of Wnt/β-catenin, Notch, and related pathways, enabling cells to evade chemotherapy-induced cytotoxicity (14, 107). Furthermore, EMT transcription factors upregulate ABC transporter genes such as ABCB1 and ABCC1, increasing drug efflux; for example, Twist1 binds the ABCB1 promoter in colorectal cancer, driving irinotecan export and resistance (108, 109). By modulating the expression of apoptotic regulators, EMT factors further inhibit drug-induced cell death (110). In addition, EMT promotes an immunosuppressive TME recruiting regulatory immune cells and upregulating checkpoint molecules (e.g., PD-L1), creating a feed-forward loop that sustains resistance to both cytotoxic agents and immunotherapy (111, 112).

### 3.4 The role of the gut microbiota

In gastrointestinal (GI) cancers, the gut microbiota profoundly modulates the metabolism of chemotherapeutic agents, thereby influencing therapeutic efficacy. For example, bacterial β-glucuronidase (GUS) can hydrolyze the inactive metabolite SN-38G into the toxic compound SN-38, leading to severe diarrhea (113). Fusobacterium nucleatum has been shown to activate the TLR4/MyD88 signaling pathway, induce reactive oxygen species (ROS) production, and attenuate DNA damage, thereby promoting chemoresistance (114). Conversely, certain microbes enhance responses to immunotherapy: Akkermansia muciniphila increases CCL5^+^CD8^+^ T-cell infiltration, thereby potentiating the efficacy of PD-1/PD-L1 immune checkpoint blockade and improving clinical outcomes (115). Moreover, microbial metabolites such as short-chain fatty acids and tryptophan-derived catabolites exert potent immunomodulatory effects (116, 117). Collectively, these findings highlight the gut microbiota as a dynamic and therapeutically targetable regulator of treatment responses in GI cancers. Interventions such as fecal microbiota transplantation or engineered bacteria–based delivery strategies hold promise for reshaping the immune microenvironment and effectively reversing tumor drug resistance (118–120).

### 3.5 Emerging technologies reveal new mechanisms

The introduction of single-cell sequencing, liquid biopsy with circulating tumor DNA (ctDNA), patient-derived organoid models, and artificial intelligence has brought about a multi-dimensional era for gastrointestinal tumor resistance research (121, 122). In colorectal cancer, single−cell RNA sequencing (scRNA-seq) has revealed a pronounced expansion of LGR5^+^ cancer stem cells following oxaliplatin treatment, with resistance maintained via Wnt/β-catenin and Notch signaling. Single-cell DNA sequencing (scDNA-seq) permits dynamic monitoring of clonal composition pre- and post-treatment for gastric cancer, with selection for TP53 and APC mutant upon chemotherapeutic challenge—offering a genetic explanation for acquired drug resistance (123–127). Liquid biopsy, analyzing ctDNA, enables non-invasive detection of resistance-driving alterations (genetic, epigenetic) and dynamic monitoring of clonal evolution, serving as a crucial tool for real-time drug sensitivity assessment and early intervention (128). Detection of KRAS/NRAS mutations in colorectal cancer ctDNA, often emerging months before radiographic progression, provides actionable insights into evolving drug sensitivity and resistance, enabling timely therapeutic adjustments. The combination of patient-derived organoid (PDO) models with *in vitro* immune cell co-culture systems establishes a powerful functional platform for validating tumor sensitivity to immunotherapy. These systems faithfully recapitulate key components of the tumor-immune microenvironment—such as tumor-associated fibroblasts, dendritic cells, macrophages, and cytotoxic T lymphocytes—and enable direct testing of how interventions targeting TGF-β, IDO1, or CSF1R can reverse immune suppression and restore anti−tumor cytotoxicity. Furthermore, generative or graph-based AI modeling approaches can simulate evolutionary trajectories of resistant subclones under immunotherapy pressure, enabling dynamic, adaptive planning of combination regimens (e.g. ICB + HDAC inhibitors or ICB + autophagy blockers) tailored to expected resistance mechanism.

Although such tools offer unmatched resolution for mapping multi-dimensional mechanisms of resistance, issues with data standardization, cost, and data complexity arise. Data sharing and harmonization are needed future initiatives, which must be targeted toward translating laboratory data into clinical applications more rapidly.

## 4 Clinical implications and mechanism-based therapeutic strategies

### 4.1 Mechanism-driven intervention approaches

Deeper insights into molecular and immunological drivers of resistance are spurring the development of targeted and immunotherapeutic interventions, evaluated both preclinically and clinically. (**Table 1**) One promising example is the KRAS G12C inhibitor sotorasib, which covalently binds the mutant cysteine in a hidden pocket of KRAS, traps the protein in its inactive GDP-bound state, and effectively quenches aberrant MAPK signaling in G12C-mutant colorectal tumors (129). To overcome efflux mediated by transporters, tariquidar, a drug inhibitor of P-gp, has been combined with nanoparticle-encapsulated paclitaxel. In gastric cancer models, it is shown to significantly increase intracellular paclitaxel concentration and restore sensitivity to taxane therapy (130–132). Concomitant efforts toward restoration of apoptosis pathways are Smac mimetics, which neutralize inhibitor-of-apoptosis proteins (IAPs) for reactivation of caspase-dependent cell death, and Bcl-2 antagonist venetoclax, which disassembles mitochondrial prosurvival defenses—both with efficacies shown in preclinical models of reversal of apoptosis blockade (38, 39, 133). Targeting the tumor stroma, TGF-β receptor blockade (e.g., galunisertib) dampens CAF activation, reduces collagen deposition, and softens the ECM. This not only enhances chemotherapy delivery and efficacy but also alleviates immunosuppression within the TME, potentially improving response to immunotherapy (85, 86, 134, 135). Reversing epithelial–mesenchymal transition (EMT) represents another avenue: the small−molecule reversine inhibits Twist1 nuclear translocation, reestablishing 5-fluorouracil sensitivity in colorectal cancer cells by restoring epithelial characteristics (109, 136, 137). Epigenetic resistance can be addressed by combining the DNA methyltransferase inhibitor azacitidine with the HDAC inhibitor panobinostat, which together reverse MGMT promoter hypermethylation, reactivate pro-apoptotic gene expression, and augment chemotherapy response in colorectal models (55, 56, 138). Lastly, simultaneous targeting of autophagy and endoplasmic reticulum stress—using hydroxychloroquine to block autophagosome–lysosome fusion and GSK2606414 to inhibit the PERK/eIF2α axis—has been shown to synergistically overcome autophagy-dependent resistance phenotypes (67, 110, 111).

**Table 1:** Mechanism-based therapeutic strategies table.

Intervention strategy	Representative agents	Clinical stage	Gastrointestinal tumor type	References
Targeted inhibition	Sotorasib	Phase III	Colorectal carcinoma	(120)
Efflux pump inhibition	Tariquidar (ABCB1 inhibitor)	Phase II	Gastric carcinoma	(121)
Apoptosis reactivation	Venetoclax	Phase I/II	Hepatocellular carcinoma	(37, 38)
DNA methyltransferase inhibitor + HDAC inhibitor	Azacitidine + Panobinostat	Phase II	Colorectal carcinoma	(55, 56)
GPX4 inhibition	Sulfasalazine (SASP)	Preclinical	Gastric carcinoma	(70–72)
TGF-β receptor blockade	Galunisertib	Phase II	Pancreatic carcinoma	(81, 82, 125)
Fecal microbiota transplantation (FMT)	–	Phase I	Melanoma (across tumor types; extrapolatable to GI cancers)	(111–113)

### 4.2 Prospects for personalized precision therapy

Drug resistance in gastrointestinal tumors reflects a heterogeneous and adaptive network of molecular and microenvironmental alterations. Personalized precision medicine aims to “tailor” therapeutic strategies to each patient’s unique resistance landscape. Integrating single−cell transcriptomics with spatial metabolomics enables precise mapping of resistant clones and their metabolic niches—for example, co−localization of KRAS−mutant subpopulations with cancer−associated fibroblasts in colorectal cancer suggests focal FAK inhibition, whereas lactate−rich microdomains in gastric cancer may be targeted via MCT1 blockade. Liquid biopsy (ctDNA, exosomes) provides real-time monitoring of drug sensitivity and resistance dynamics, enabling early detection of recurrence and data-driven treatment adaptation (139).Mechanism-based combinatorial strategies integrating immunotherapy are emerging: e.g.,triplet therapies combining KRAS inhibitors, immune checkpoint blockade (ICB), and epigenetic modulators (like HDAC inhibitors that enhance tumor antigen presentation) show synergistic efficacy. In recent years, multiple trials, including the KRYSTAL-1 trial (NCT03785249), have demonstrated that triplet therapy can effectively reverse acquired resistance in gastrointestinal tumors (140–143). Dual targeting of epithelial mesenchymal transition and autophagy (e.g., Twist1 inhibitors plus hydroxychloroquine) can dismantle the survival networks of cancer stem like cells and re-sensitize tumors to conventional chemotherapy.AI-driven sensitivity prediction is a key strategy to overcome drug resistance. For instance, multimodal AI platforms integrate genomic features (such as IFN-γ response genes), digital pathology images, and ex vivo organoid drug screening data (144, 145), generating patient-specific immunotherapy sensitivity scores with predictive accuracy superior to single biomarkers like PD-L1 IHC. Studies have shown that such AI-based prediction models exhibit immense potential in oncology (146, 147), providing powerful tools for personalized treatment decisions. Rigorous clinical validation of these approaches, with attention to safety, feasibility, and cost-effectiveness, will be essential. Ultimately, the goal of personalized precision therapy is not only to surmount therapeutic resistance but also to preserve patient dignity and hope throughout the treatment journey.

## 5 Conclusion and future perspectives

Drug resistance in GI malignancies represents a complex “survival race,” driven by tumor cell-intrinsic mechanisms (genetic, epigenetic) and extrinsic factors (microenvironmental crosstalk, immune evasion) to evade cytotoxic, targeted, and immunotherapies. These mechanisms operate not in isolation but as a dynamic, interconnected network, collectively enabling tumor persistence and progression under therapeutic pressure.

Interdisciplinary technologies (spatial/single-cell omics, liquid biopsy, organoids, AI) are deciphering this complexity, enabling more precise mapping of resistance mechanisms (including immune evasion) and facilitating drug sensitivity profiling for improved clinical decisions. Nevertheless, there are substantial issues, including integration of data, standardization, cost, and translation into everyday practice.

Prospects for future research include high-resolution, spatiotemporal mapping of resistance niches; deep learning platforms predicting adaptive responses and optimizing real-time treatment regimens; proactive intervention approaches that preempt and countermand resistance; and rational reengineering of the tumor microenvironment for dismantling protective niches. Leveraging these intelligent tools and patient-centric strategies-particularly those integrating immunotherapy insights and drug sensitivity analysis-holds promise for transforming drug resistance into a navigable challenge, leading to sustained remission and enhanced patient outcomes.
